# Cytomegalovirus host receptor expression in the human fetal inner ear

**DOI:** 10.1371/journal.pone.0320605

**Published:** 2025-03-31

**Authors:** Lucia C. M. Grijpink, Wouter H. van der Valk, Edward S. A. van Beelen, John C. M. J. de Groot, Heiko Locher, Ann C. T. M. Vossen

**Affiliations:** 1 Leiden University Center for Infectious Diseases (LUCID), Medical Microbiology and Infection Control, Leiden University Medical Center, Leiden, The Netherlands; 2 Department of Otorhinolaryngology and Head & Neck Surgery, Leiden University Medical Center, Leiden, The Netherlands; 3 The Novo Nordisk Foundation Center for Stem Cell Medicine (reNEW), Leiden University Medical Center, Leiden, The Netherlands; Stanford University, UNITED STATES OF AMERICA

## Abstract

Fetal infection with human cytomegalovirus (hCMV) can cause sensorineural hearing loss and vestibular impairment, yet its pathogenesis remains unclear. This study aims to identify potential target cell types of hCMV in the human fetal inner ear. Viral particles use several envelope glycoproteins to enter target cells, including the pentameric complex, the trimeric complex and glycoprotein B. Platelet-derived growth factor receptor alpha (PDGFRA) serves as the receptor in fibroblasts, neuropilin-2 (NRP2) in epithelial, endothelial and dendritic cells as well as in leukocytes. Upon binding of these glycoproteins, glycoprotein B initiates membrane fusion which is proposed to be mediated by EGFR. When and where these proteins are expressed in the fetal inner ear during development is unknown. To address this, expression patterns of PDGFRA, NRP2 and EGFR were investigated in human fetal inner ear tissue using single-nucleus RNA sequencing data (first trimester: N = 2) and immunohistochemistry (first trimester: N = 6, second trimester: N = 5). *PDGFRA* gene and protein expression was detected in mesenchymal cells, NRP2 protein expression in epithelial cells and endothelial cells, and *EGFR* gene and protein expression in both epithelial cells and mesenchymal cells. Notably, all three receptors were present in tissue from the first and second trimesters. In conclusion, hCMV host receptors PDGFRA, NRP2 and EGFR are expressed in mesenchymal, epithelial and endothelial cells within the cochlea and vestibular organs during the first and second trimesters. These cell types may serve as targets for hCMV infection of the fetal inner ear.

## Introduction

Human cytomegalovirus (hCMV), a member of the *Herpesviridae* family and *Betaherpesvirinae* subfamily, is a double-stranded DNA virus with a genome size of approximately 235 kb, making it one of the largest known viral genomes. This virus is the most common cause of congenital infection worldwide, with a birth prevalence ranging from 0.5% in high-income countries up to 6% in low-income countries [1,2]. Congenital hCMV infection can cause permanent disabilities, such as cognitive and motor developmental delay, and it is the leading cause of non-genetic congenital sensorineural hearing loss (SNHL). About 12.6% of congenitally infected children will develop SNHL [3]. Furthermore, congenital hCMV infection can cause vestibular impairment with an incidence that matches or even exceeds that of SNHL [4,5]. However, the pathogenesis of hCMV-induced inner ear dysfunction has not yet been elucidated, which hampers the development of preventive and therapeutic strategies.

Uncovering the pathogenesis of inner ear infection with CMV has been the subject of animal studies in mice, guinea pigs and rhesus macaques [6–8]. These studies have demonstrated several effects of CMV infection of the inner ear, such as increased blood-labyrinth permeability, influx of immune cells, and apoptosis of neurons [9–11]. However, the results of these animal studies cannot be directly extrapolated to the human situation, as CMV is species specific. Even though mice display typical symptoms such as hearing loss and microcephaly, the viruses used in animal studies exhibit different biological characteristics than hCMV [12,13]. Another caveat to the commonly used mouse model is that transplacental transmission - the route of hCMV transmission in humans - does not occur and therefore the virus must be injected intracranially or -peritoneally into newborn mice to mimic congenital infection. Thus animal models have proven beneficial, but they do not entirely reflect the complexity of human CMV infection.

Human postmortem studies have been performed on the temporal bones of severely affected fetuses and newborns, revealing infection of inner ear cells, particularly the stria vascularis [14–17]. As these cases showed signs of advanced infection on fetal ultrasound, they do not provide insights into the initial stages of hCMV pathogenesis. This initial phase of infection and first damage may already have taken place in the first trimester, since congenital infection in this period results in significantly more permanent symptoms than infection in the second or third trimesters [18–20]. For a comprehensive understanding of the pathogenic mechanism of permanent inner ear dysfunction due to hCMV infection, emphasis should therefore be placed on early developmental stages.

**Fig 1:**
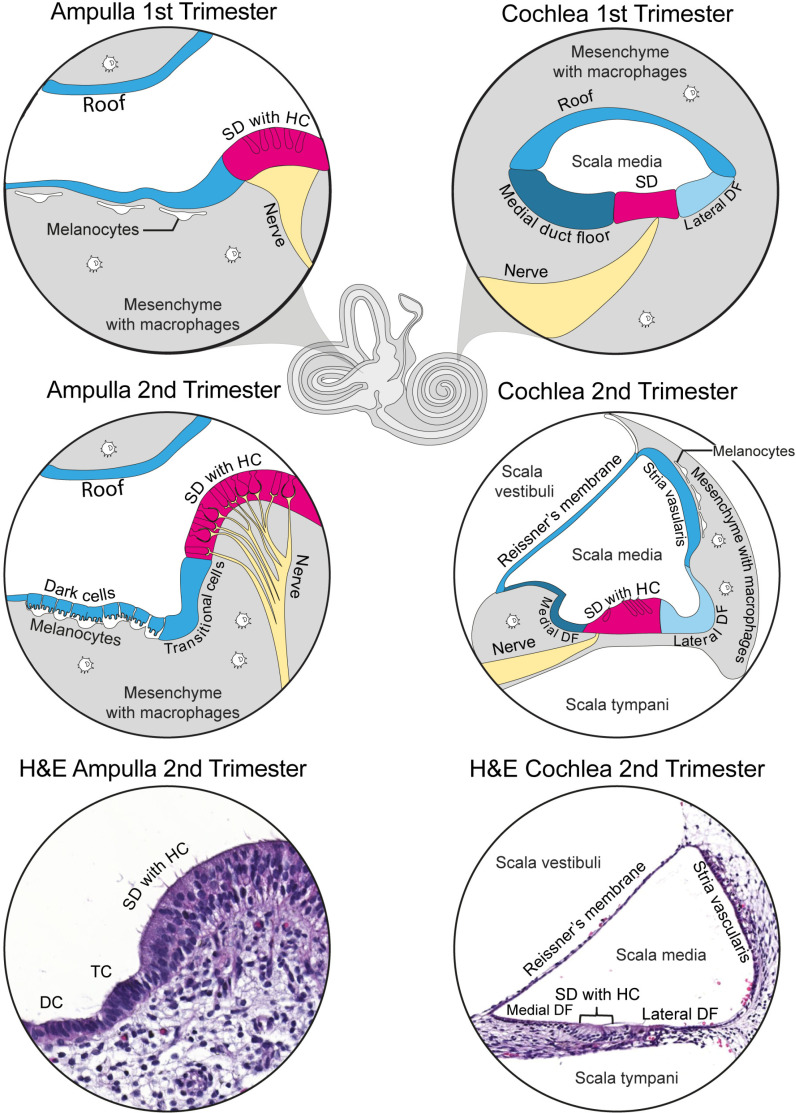
Illustration of the human inner ear. The main cell types of the cochlea and ampulla are depicted with the corresponding H&E stainings. The ampulla is from a FW10.5 fetus and the scala media is from a FW14.2 fetus. The sensory domain (SD) is depicted in magenta and is composed of the supporting cells and hair cells (HC). In the first trimester, the cochlea does not yet contain hair cells, so its sensory domain consists only of supporting cells. Non-sensory epithelial cells are shown in blue. In the cochlea, these cells form the medial and lateral duct floor (DF) and the roof, which will develop into Reissner’s membrane and the stria vascularis. Also in the vestibular organs, the roof is lined with non-sensory epithelium. Additionally, the dark cells (DC) and transitional cells (TC)(except in the saccule) contain non-sensory epithelium.

The development of the human inner ear begins early in the embryonic period with formation taking place in the first trimester and maturation in the second trimester [21,22]. The inner ear consists of the cochlea and the vestibular organs, as depicted in Fig 1. The cochlea is the organ of sound perception and contains three fluid-filled tubes which spiral around a central axis, namely the scala vestibuli, scala media and scala tympani. The scala media is lined with sensory epithelium (the sensory domain), which contains the hair cells and supporting cells. The roof, medial and lateral duct floor contain the non-sensory epithelial cells (Fig 1). As sound waves are transmitted to the cochlea, mechanical stimulation of the hair cells results in the generation of a compound action potential that is transported to the brainstem via the neurons [23]. The vestibular organs consist of three ampullae, the saccule and the utricle and sense balance and spatial orientation. These organs also contain sensory domain with supporting cells and hair cells that depolarize upon mechanical stimulation. The vestibular roof is lined with non-sensory epithelium. Also the dark cells and transitional cells contain non-sensory epithelium (Fig 1).

Ex-vivo infection studies on human fetal inner ear tissue are not feasible due to the difficulty of obtaining adequate amounts of this delicate, limited tissue with consistent quality. Instead, it is possible to investigate another critical aspect of CMV infection in this tissue: the host receptor expression.

hCMV is known to have a broad tropism [24,25]. Viral glycoproteins are a major component of the lipid envelope and several of them have been identified to play a key role in binding to the target cell, using host receptors (S1 Fig). Firstly, *in vitro* studies in human cell lines have shown that the hCMV envelope glycoproteins H (gH), L (gL) and O (gO) form the trimeric complex, which binds to platelet-derived growth factor receptor alpha (PDGFRA) to enter fibroblasts [26,27]. Secondly, the pentameric complex is composed of gH, gL, UL128, UL130 and UL131A and, via binding to neuropilin-2 (NRP2), mediates entry into endothelial, epithelial and dendritic cells, as well as leukocytes [28–31]. Finally, it has been demonstrated that upon binding of the trimeric and pentameric complex to the host cell, glycoprotein B (gB) is triggered and consequently initiates the fusion of the viral envelope and the plasma membrane of the host cell [32–34]. Epidermal growth factor receptor (EGFR) has been proposed as the ligand of this so-called ‘fusion protein’ [35]. Identifying the cell types in the fetal inner ear that express these receptors is important to understand the pathogenesis of hCMV-induced inner ear dysfunction. To date, no data is available on the human inner ear in the first trimester.

This study investigated the gene and protein expression of PDGFRA, NRP2, and EGFR in the human fetal inner ear, combining gene expression data from a previously published single-nucleus RNA sequencing (snRNAseq) dataset with protein expression data obtained through immunohistochemistry (IHC). Our results demonstrate the presence of all three host receptors within the cochlea and vestibular organs, contributing to our understanding of hCMV inner ear pathogenesis.

## Materials and methods

### Ethics statement

The use of human embryonic and fetal tissue was in accordance with Dutch legislation (Fetal Tissue Act, 2021; Embryo Act, 2021) and the WMA Declaration of Helsinki guidelines. Approval for the project was granted by the Medical Ethics Committee Leiden The Hague Delft (protocol registration number B18.044). Written informed consent was obtained from the women who underwent elective termination of pregnancy in accordance with the Guidelines on the Provision of Fetal Tissue as outlined by the Dutch Ministry of Health, Welfare, and Sport (revised version, 2018). This study did not include minors. Recruitment for this study took place between January 11 and August 2, 2019.

### Tissue collection

Fetal tissue was obtained after elective termination of pregnancy by uterine vacuum aspiration. Duration of pregnancy (gestational age) was determined by ultrasound scan with a standard error of 2 days. Fetal age is calculated by subtracting two weeks from the gestational age. Fetal age is expressed as fetal weeks (x) plus number of days (y) (FWx.y).

### Single-nucleus RNA sequencing and data analysis

The snRNAseq data of the human inner ear used in this study were originally generated by van der Valk et al. [36] and included both adult and fetal data. For our current analysis, we specifically reused the sequencing data from two fetal inner ear timepoints: fetal week 7 (FW7.5) and fetal week 9 (FW9.2).

In brief, the original study collected data as follows: fresh human fetal inner ear tissues were collected, washed in phosphate-buffered saline (PBS), and dissected. The tissues were stored overnight at 4°C in RNAlater solution (AM7020, Invitrogen, USA). Within 16 hours of collection, the tissues were processed into single nuclei suspensions. The single-nucleus gene expression libraries were prepared using the 10x Genomics Chromium platform, employing the Chromium Next GEM Single Cell 3′ Library & Gel Bead Kit v3.1 and the Chromium Next GEM Chip G Single Cell Kit (10x Genomics).

The Cell Ranger mkfastq (10x Genomics) was utilized to generate FASTQ files from the raw sequencing reads. The demultiplexed FASTQ files were processed through the 10x Genomics Cell Ranger 6.1.0 pipeline (available at http://support.10xgenomics.com/), aligning the reads to the human reference genome GRCh38, including both intronic and exonic regions. Gene expression levels were quantified by counting unique molecular identifiers (UMIs) detected per cell, resulting in filtered gene expression matrices.

As mentioned in the original paper by Van der Valk et al., the integrated datasets underwent quality control and were used for clustering visualization using Uniform Manifold Approximation and Projection (UMAP) techniques. Cell identity assignment was performed through differential expression analysis and manual assessment with cell type-specific marker genes using recently published single-cell datasets of the mouse inner ear [37–41]. These annotations were validated by immunohistochemistry (IHC) to characterize the subpopulations within the integrated inner ear dataset. Quantitative gene expression and gene expression patterns of host cellular receptors were plotted using the R package ggplot2 [42]. Scripts used for the human inner ear dataset are available at https://github.com/OtoBiologyLeiden/.

### Immunohistochemistry

Fetal tissue from both the first (up to FW10.6, N = 6) and second trimesters (from FW11.0 up to FW24.6, N = 5) was used: FW7.4, FW8.0, FW8.6, FW9.6, FW9.6, FW10.5, FW11.5, FW12.6, FW14.2, FW14.4, FW16.5. The inner ear tissue was processed as previously described [43]. Inner ear tissue was dissected directly after vacuum aspiration, washed in phosphate-buffered saline (PBS), transferred to 4% buffered formaldehyde and transported on ice. The tissue was fixed for 12-72 hours at 4°C. Inner ears from FW12 and older were decalcified in 10% EDTA.2Na (Sigma‐Aldrich, USA) in distilled water (pH 7.4) for 1–3 weeks at 4°C. After dehydration in an ascending ethanol series (70-99%) and clearing in xylene, the tissue was embedded in paraffin and cut into 5 µm sections using an HM 355 S rotary microtome (Thermo Fisher Scientific, USA). Sections were transferred to silane-coated glass slides and air-dried overnight at room temperature.

Tissue sections were deparaffinized in xylene and rehydrated in a descending ethanol series (96–70 –50%). Routine hematoxylin and eosin (H&E) staining was applied every 10 sections to localize the anatomical areas and cell types of interest in the tissue, using hematoxylin (40859001, Klinipath, Netherlands) and eosin (40829002, Klinipath, Netherlands). For IHC, heat-induced antigen retrieval was applied using a 10 mM sodium citrate buffer (pH adjusted to 6.0 using hydrochloric acid). To prevent non-specific binding of antibodies, sections were immersed in a blocking buffer (1% bovine serum albumin (A3059-50G, Sigma-Aldrich, USA), 5% normal donkey serum (ab7475, Abcam, UK), 0.05% Tween-20 (8.22184, Merck Group, Germany) in PBS) for 30 minutes. The sections were incubated overnight at 4°C with primary antibodies or corresponding isotype controls (Table 1). A combination of antibodies against host receptors and inner ear cell type markers was used. A second blocking step was applied for 10 minutes, after which the sections were incubated with secondary antibodies for 1 hour at room temperature (Table 2). Nuclei were stained with DAPI (D3571, Invitrogen, USA) for 5 minutes.

**Table 1:** Overview of primary antibodies and isotypes.

Antibody	Host	Company	Catalog-number	RRID	Dilution
	**Primary antibodies**				
AIF1	Goat	Novus Biologicals	NB 100-1028	AB_521594	1:125
CD68	Rabbit	Abcam	ab213363	AB_2801637	1:1000
EGFR	Rabbit	Thermo Fisher Scientific	PA1-1110	AB_326079	1:300
MLANA	Rabbit	Novus Biologicals	NBP1-30151	AB_1987285	1:200
MYO7A	Mouse	DSHB	138-1-s	AB_2282417	3:10
NRP2	Mouse	Santa Cruz Biotechnology	sc-13117	AB_628044	1:200
PDGFRA	Goat	Novus Biologicals	AF-307-NA	AB_354459	1:100
PECAM1	Rabbit	Novus Biologicals	NB100-2284	AB_10002513	1:100
TUBB3	Mouse	Abcam	ab7751	AB_306045	1:500
TUBB3	Rabbit	Abcam	ab18207	AB_444319	1:200
	**Isotype controls**				
IgG	Goat	Novus Biologicals	NB410-28088	AB_1853319	1:250
IgG	Mouse	Thermo Fisher Scientific	08-6599	AB_2532952	Undiluted
IgG	Rabbit	Thermo Fisher Scientific	08-6199	AB_2532942	Undiluted

**Table 2:** Overview of secondary antibodies.

Antibody	Conjugate	Company	Catalog-number	Dilution
Donkey anti-goat IgG	Alexa Fluor 488	Invitrogen	A11055	1:1000
Donkey anti-goat IgG	Alexa Fluor 680	Invitrogen	A21084	1:500
Donkey anti-mouse IgG	Alexa Fluor 488	Invitrogen	A21202	1:500
Donkey anti-mouse IgG	Alexa Fluor 594	Invitrogen	A21203	1:1000
Donkey anti-mouse IgG	Alexa Fluor 680	Invitrogen	A10038	1:1000
Donkey anti-rabbit IgG	Alexa Fluor 488	Invitrogen	A21206	1:500
Donkey anti-rabbit IgG	Alexa Fluor 594	Invitrogen	A21207	1:1000
Donkey anti-rabbit IgG	Alexa Fluor 680	Invitrogen	A10043	1:500

The sections were mounted in Prolong Gold antifade reagent (P36934, Invitrogen, USA) and affixed with a coverslip. Washing between the steps was performed using a washing buffer (0.05% Tween-20 in PBS).

Negative controls consisted of sections where primary antibodies were replaced by matching isotype controls. Positive controls consisted of sections of human tissue samples known to express the specific antigen. DOI: dx.doi.org/10.17504/protocols.io.6qpvr9qzzvmk/v1

### Imaging and interpretation

H&E sections were imaged using a 3DHistech Pannoramic 250 scanner and viewed with SlideViewer software (3DHISTECH, Hungary). Immunostained sections were imaged with a Leica SP8 confocal laser scanning microscope and a Leica DM5500 B microscope, operating under Leica Application Suite X microscope software (LAS X, Leica Microsystems, Germany). Maximal projections were obtained from image stacks, and brightness and contrast adjustments were performed with Fiji (ImageJ 1.53c). Immunohistochemical staining for each host receptor was performed in at least 4 inner ears per trimester. The immunohistochemical images represent inner ear tissue collected from separate tissues within the same trimester.

A random sample of the immunostained sections was independently assessed by two blinded co-authors (JdG, HL). The results were compared to the first author’s interpretation to ensure objective assessment. If the results were discordant, they were discussed with the co-authors to reach a consensus.

## Results

### PDGFRA, NRP2 and EGFR genes are expressed in the first trimester

**Fig 2:**
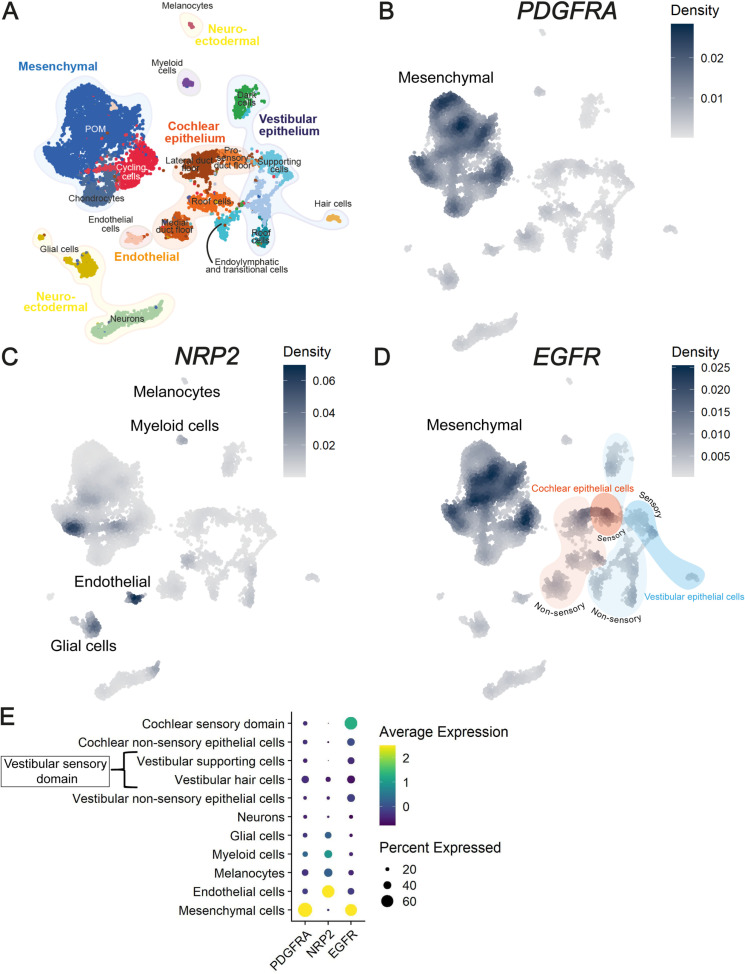
snRNAseq data of FW7.5 and FW9.2 human fetal inner ears. (A) Uniform Manifold Approximation and Projection (UMAP) plot with cell type annotations. (B–D) Feature plots showing gene expression of *PDGFRA, NRP2* and *EGFR based on the kernel density*. (D) cochlear epithelial cells are indicated in orange (dark: sensory domain, light: non-sensory epithelium) and vestibular epithelial cells are indicated in blue (dark: sensory domain, light: non-sensory epithelium). (E) Dot plot showing standardized gene expression of *PDGFRA, NRP2* and *EGFR* in relevant inner ear cell types.

Expression of *PDGFRA, NRP2* and *EGFR* genes was analyzed using recently published snRNAseq data in human fetal inner ears of FW7.5 and FW9.2 [36]. The data collection process for the original dataset is outlined in the Methods section, and findings on specific cellular host receptors are displayed in Fig 2. The inner ear anatomy and its cell types are depicted in Fig 1.

Different inner ear cell types were identified in the snRNAseq data, as shown in the UMAP (Fig 2A). It was possible to distinguish between cochlear and vestibular cells in the case of sensory domains and non-sensory epithelial cell populations, but this distinction could not be made for other cell types. Therefore, neurons, melanocytes, mesenchymal cells, myeloid cells and endothelial cells are not defined as cochlear or vestibular (Table 3). The population of hair cells was of vestibular origin, as cochlear hair cells develop later than vestibular hair cells and only start to form at FW10.

**Table 3:** RNA and protein expression of hCMV host receptors in the inner ear cell types.

	PDGFRA			NRP2			EGFR		
	1st trimester		2nd trimester	1st trimester		2nd trimester	1st trimester		2nd trimester
	RNA	Protein	Protein	RNA	Protein ^c^	Protein	RNA	Protein	Protein
**Cochlear**									
Sensory domain	–	–	–	–	+/-	+/-	++	+	+
Non-sensory epithelial cells	–	–	–	–	+/-	+	+	+/- ^d^	+/-
**Vestibular**									
Sensory domain	+	–	–	–	+/-	+/-	+	+	+
Non-sensory epithelial cells	–	–	–	–	+/-	+	+	+	+
**Unknown** ^a^									
Neurons	–	–	–	–	–	–	–	–	–
Myeloid cells (containing macrophages)	–	–	–	+	+	+	–	+	+
Melanocytes	+	N/A ^b^	–	+	N/A	+/-	–	N/A	+
Endothelial cells	–	–	–	++	+	+	–	–	–
Mesenchymal cells	++	+	+	–	+	+	++	+	+

^a^It was possible to distinguish between cochlear and vestibular cells in the case of sensory domains and non-sensory epithelial cell populations, but for other cell types it was not possible to make this distinction. Therefore, neurons, melanocytes, mesenchymal cells, myeloid cells and endothelial cells are not defined as cochlear or vestibular.

^b^Melanocytes were not observed in the first trimester.

^c^The punctate staining pattern posed challenges in identifying the exact cells with immunostaining. Nonetheless, NRP2 staining was noticeable in epithelial cells.

^d^EGFR was not expressed in the greater epithelial ridge and lesser epithelial ridge.

The feature plots depict the gene expression of *PDGFRA, NRP2* and *EGFR* in fetal inner ear tissue (Fig 2B–2D), which is also shown in the dot plot (Fig 2E). An extended version of this dot plot is included in the supplementary material (S2 Fig), including known marker genes in cochlear and vestibular cell types. *PDGFRA* is expressed in the population of mesenchymal cells (Fig 2B and 2E and S2 Fig). Gene expression of *NRP2* predominantly localizes to endothelial cells, myeloid cells and melanocytes (Fig 2C and 2E and S2 Fig). There is also a sub-population of the mesenchymal cells that expresses NRP2. This sub-population was previously reported to be associated to the otic capsule, which is the bony structure surrounding the inner ear [36]. *EGFR* gene expression is predominantly found in the cochlear sensory domain and mesenchymal cells. Additionally, there is a lower level of expression in cochlear non-sensory epithelial cells, vestibular sensory and non-sensory epithelial cells (Fig 2D and 2E and S2 Fig).

### PDGFRA protein is expressed in cochlear and vestibular mesenchyme

**Fig 3:**
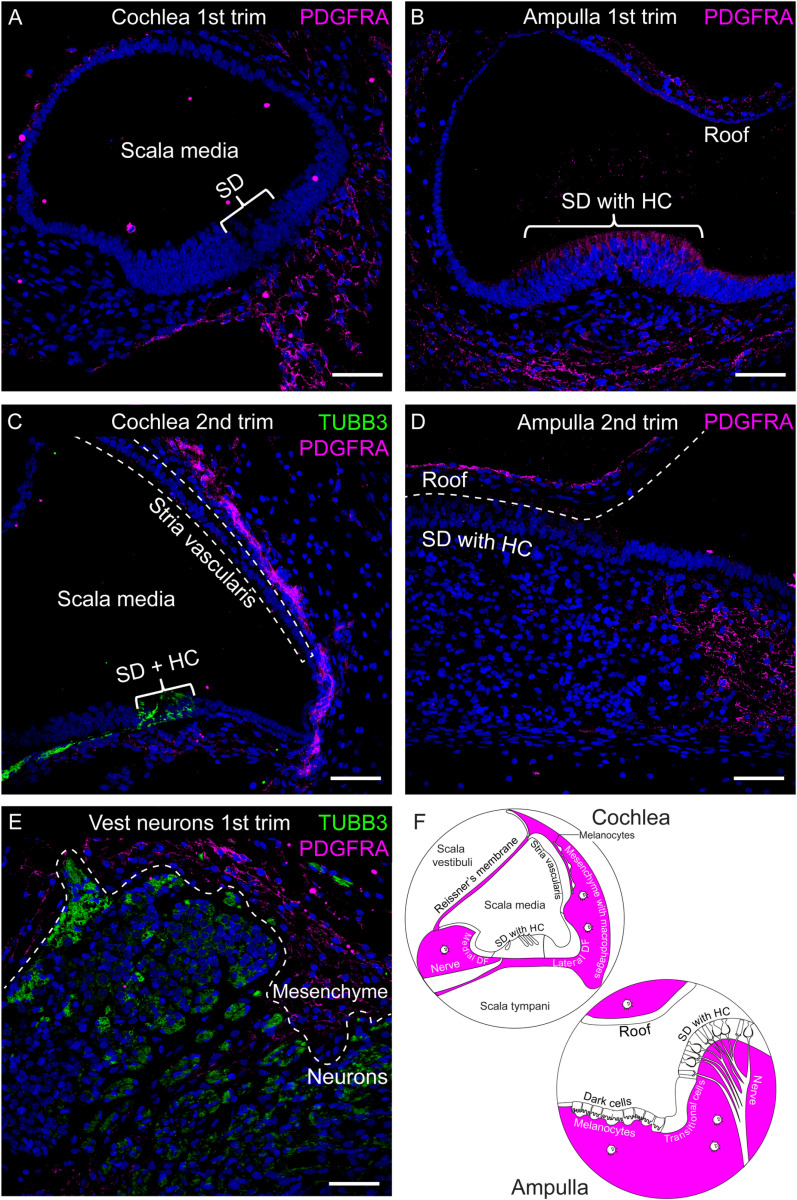
PDGFRA (magenta) protein expression in fetal human inner ear tissue from both first and second trimesters. Neurons are immunostained with tubulin beta 3 class III (TUBB3, green). Nuclei were stained with DAPI. Trim =  trimester, SD =  sensory domain, HC =  hair cell, DF =  duct floor. (A) Scala media at FW10.5, (B) Ampulla at FW8.6. The fluorescent signal seen in the sensory domain is autofluorescence, as confirmed by isotype control. (C) Scala media at FW14.2, (D) Ampulla at FW14 with a collapsed roof, demarcated by the dotted line, (E) Vestibular ganglion at FW9.6, (F) Illustration of the anatomical locations of PDGFRA expression (magenta) representative for immunofluorescent staining of 11 tissues. Scale bar =  50 μm.

Immunohistochemical examination detected PDGFRA^ +^ cells in both the first and second trimesters (Fig 3A–3F). A distinct expression pattern was observed, with immunofluorescent signal exclusively in mesenchymal cells in the cochlea and vestibular organs. Additionally, PDGFRA^ +^ cells were found in the mesothelial cell layer of Reissner’s membrane, facing the scala vestibuli (S3 Fig) and in the mesenchyme surrounding the vestibular neurons (Fig 3E). The expression pattern did not differ between the first and second trimesters. The sensory domain showed fluorescent signal that looked different from the membranous staining pattern in the mesenchymal cells (Fig 3B). Immunofluorescent staining using isotype control confirmed that this was autofluorescent signal (S4 Fig).

### NRP2 protein is expressed in macrophages, epithelial, endothelial and mesenchymal cells in the cochlea and vestibular organs

**Fig 4:**
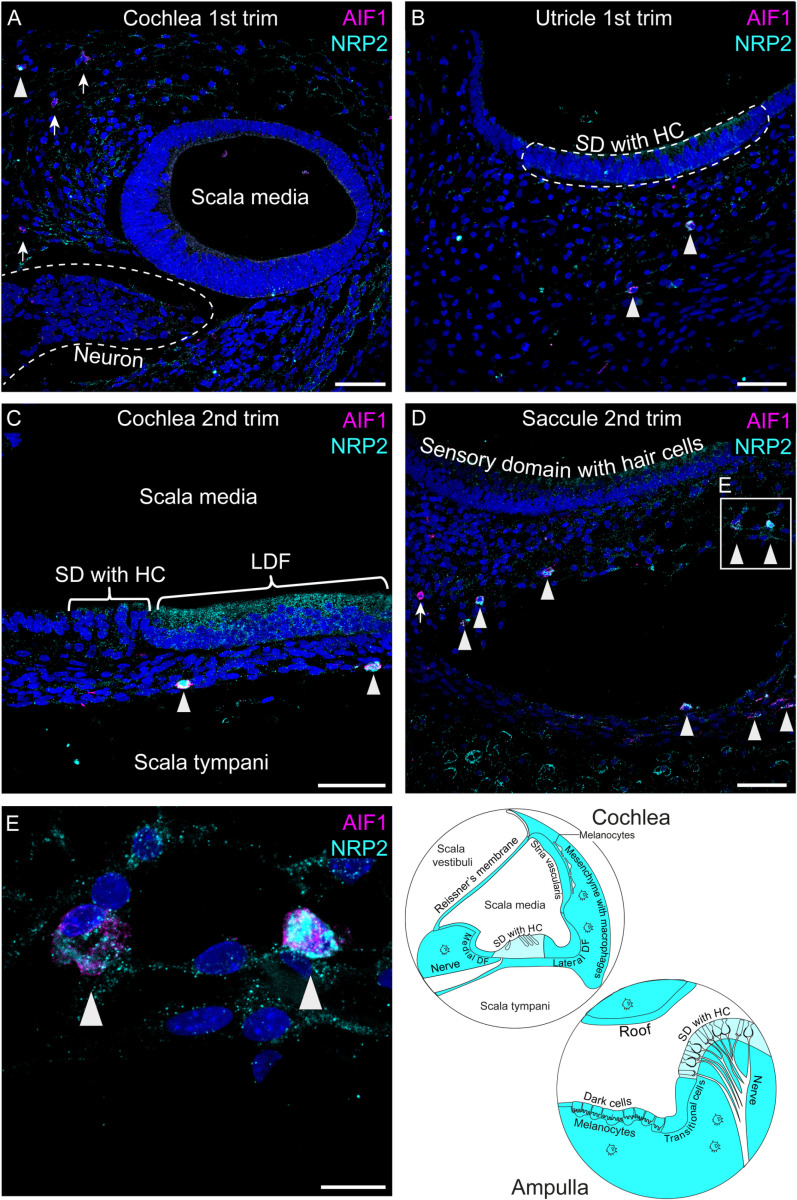
NRP2 (cyan) protein expression in fetal human inner ear tissue from both first and second trimesters. Macrophages are immunostained with AIF1 (magenta). Arrowheads indicate AIF1^ + ^NRP2^ +^ cells. Arrows indicate AIF1^ + ^NRP2^-^ cells. Nuclei were stained with DAPI. Trim =  trimester, SD =  sensory domain, HC =  hair cell, DF =  duct floor. (A) Scala media at FW8.6, (B) Utricle at FW8.6, (C) Scala media at FW14.2, (D) Saccule at FW14.2, (E) Detail of D showing NRP2 immunostaining of macrophages, (F) Illustration of the anatomical locations of NRP2 expression (cyan) representative for immunofluorescent staining of 9 tissues. Scale bar in images A-D =  50 μm, scale bar in image E =  10 μm.

Immunostaining of NRP2 was present in mesenchymal cells in the first and second trimesters (Fig 4A–4F). Certain cells within the mesenchyme exhibited a more intense fluorescent signal, and these cells were also positive for allograft inflammatory factor-1 (AIF1) (Fig 4A–4E) and CD68 (S5 Fig), markers for macrophages [44]. Not all AIF1^ +^ cells showed NRP2 positivity (Fig 4A, 4B and 4D). The punctate staining pattern, clearly visible in Fig 4E, posed challenges in identifying the exact cells with immunostaining. Nonetheless, NRP2 staining was noticeable in epithelial cells (Fig 4C and S6 Fig). Because the snRNAseq data indicated gene expression in endothelial cells and to a lesser degree in melanocytes, we immunostained inner ear tissue for PECAM1, an endothelial marker, and melanocyte marker MLANA (S6 Fig). Endothelial cells showed NRP2 protein expression in both the first and second trimesters (S6A Fig). In the second trimester, some melanocytes displayed the typical punctate NRP2 staining, though not all (S6B Fig). Thus, NRP2 protein expression is seen in macrophages, epithelial cells, endothelial cells and to a lesser extent in the mesenchymal cells in both the cochlea and vestibular organs, in the first and second trimesters.

### Broad protein expression of EGFR in the cochlea and vestibular organs

**Fig 5:**
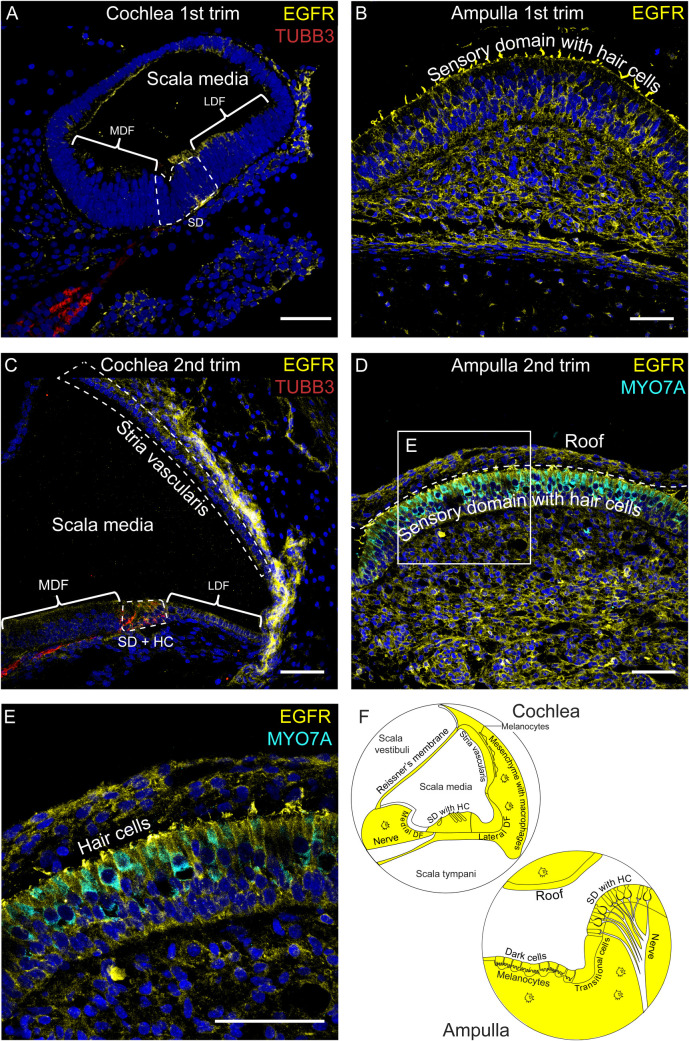
EGFR (yellow) protein expression in human fetal inner ear tissue from both first and second trimesters. Neurons are immunostained with tubulin beta 3 class III (TUBB3, red) and hair cells with myosin VIIA (MYO7A, cyan). Nuclei were stained with DAPI. Trim =  trimester, SD =  sensory domain, HC =  hair cell, MDF =  medial duct floor, LDF =  lateral duct floor. (A) Scala media at FW9.6, (B) Ampulla at FW10.5, (C) Scala media at FW14.2, (D) Ampulla at FW14.2 with a collapsed roof, demarcated by the dotted line, (E) Detail of D showing MYO7A immunostaining in the cell bodies of vestibular hair cells and EGFR immunostaining in the stereocilia, **(F)** Illustration of the anatomical locations of EGFR expression (yellow) representative for immunofluorescent staining of 11 tissues. Scale bar =  50 μm.

Consistent with the snRNAseq data, EGFR immunostaining showed a generalized expression pattern throughout the inner ear, evident as early as the first trimester (Fig 5A–5F). In the first trimester cochlea, immunofluorescent signal was visible in the sensory domain and lateral duct floor (Fig 5A), but absent in the medial duct floor. In the second trimester, EGFR protein expression was again observed in the lateral duct floor and hair cells, but remained absent in the medial duct floor (Fig 5C). Notably, vestibular stereocilia, the mechanosensory organelles of hair cells, showed immunostaining in both the first and second trimesters (Fig 5B, 5D–5E). While endothelial cells and neurons showed no EGFR expression, mesenchymal cells, including the surrounding fibrocytes of neurons, and macrophages did express EGFR.

A summary of the gene and protein expression of the host receptors per inner ear cell type is provided in Table 3.

## Discussion

In this study, we mapped the expression of host receptors essential for hCMV entry into the cells of the developing human inner ear. We showed expression of the host receptors PDGFRA, NRP2 and EGFR at both gene and protein levels in first and second trimester human cochlea and vestibular organs.

PDGFRA has been identified as the functional receptor of hCMV into fibroblasts [26]. Both PDGFRA gene and protein expression were detected in mesenchymal cells during the first and second trimesters, suggesting that these cells are susceptible to hCMV infection. NRP2 serves as the functional receptor in epithelial cells and endothelial cells [30]. In both cell types, NRP2 expression was detected in the first and second trimesters. While its role in viral entry into other cell types remains uncertain, our study revealed NRP2 expression in mesenchymal cells and macrophages. Although not essential for hCMV entry into fibroblasts, the combined presence of NRP2 and PDGFRA may increase susceptibility to viral infection [30]. Previous studies have also reported NRP2 expression in macrophages [45,46]. It is hypothesized that macrophages, capable of harboring both lytic and latent infections, play a critical role during hCMV infection [47–49].

Glycoprotein b initiates the fusion of the virus with the host cell after the initial attachment. The necessity of binding with EGFR during this process has been the subject of scientific debate [35,50]. In this study, EGFR expression was detected on a variety of cell types. Mesenchymal cells expressed EGFR, NRP2 and PDGFRA, epithelial cells and macrophages expressed NRP2 and EGFR.

The challenge of studying human fetal inner ear tissue is reflected in the paucity of literature in this area. To our knowledge, only one study has previously examined EGFR expression in the human fetal inner ear. Schwentner et al. performed IHC on fetal cochleae from three patients with Turner syndrome and four controls without genetic disorder, all during the second trimester [51]. The authors showed EGFR protein expression in the cochlea and in the mesenchymal cells surrounding the neurons as well as in the stria vascularis in FW18 and FW21. Additionally, EGFR expression was noted in Reissner’s membrane and hair cells in a fetus of FW14. Our study similarly detected EGFR expression in these cell types. Furthermore, Schwenter et al. observed the absence of EGFR expression in the greater epithelial ridge, originating from the medial duct floor, a finding corroborated by our observation of no EGFR staining in this region. Notably, their investigation did not extend to the vestibular organs. Taken together, our findings are consistent with existing literature and provide the first description of EGFR expression in the inner ear during the first trimester and in the vestibular organs during the second trimester. However, it is important to note that our study focused on the expression of these host receptors in human fetal tissue and did not include functional assays to assess their role in hCMV infection. To further investigate the pathogenetic mechanisms, *in vivo* models, such as animal and organoid models, will be essential.

Fetal infection with hCMV in the first trimester of pregnancy is associated with a significantly higher incidence of congenital and late-onset hearing loss compared to infections in later trimesters [18–20]. Our study detected host receptors that serve as primary entry proteins for hCMV, as early as FW7-8. Interestingly, we observed no obvious difference in host receptor expression between the first and second trimester. This suggests that the difference in clinical outcomes between first and second trimester hCMV infections may involve mechanisms beyond the expression of these host receptors, such as a lack of mature adaptive immune response in the fetus.

Post-mortem examination of inner ears from fetuses infected with hCMV *in utero* has revealed infection of non-sensory epithelial cells, predominantly in the stria vascularis, as well as in the Reissner’s membrane and similar structures in the vestibular organs [14–17]. Infection of hair cells and neurons was occasionally observed, sometimes potentially masked by autolysis of the organ of Corti. It was therefore hypothesized that inner ear dysfunction is not caused by direct viral damage to hair cells or neurons, but rather by damage to ion channels within the stria vascularis. Consequently, an ion imbalance may develop, ultimately resulting in improper stimulation or degeneration of hair cells [14,16]. The expression of NPR2 and EGFR in non-sensory epithelial cells, including in the developing stria vascularis in both the first and second trimesters, supports this hypothesis.

It is important to note that hCMV entry into human cells is a complex pathway involving numerous additional proteins beyond those investigated here. These include integrins [52,53], OR14i1 [54], THY1 [55,56], CD46 [57], basigin (CD147) [58], CD151 [59] and alanyl aminopeptidase (CD13) [60]. For this study, we decided to focus on the proteins that enable the initial binding of the viral particle to the host cell, as this is a crucial step prior to the activation of any subsequent proteins.

Gene expression by snRNAseq and protein expression by IHC were not always in concordance; for instance, NRP2 protein expression was observed in epithelial and mesenchymal cells, while gene expression was absent. Such discrepancies may arise from the different temporal dynamics of mRNA expression and protein synthesis measured by these techniques. Also a difference in sensitivity of the techniques used can affect the results [61].

Altogether, we have mapped the expression patterns of crucial hCMV host receptors in the developing inner ear. Hereby, we have laid the groundwork for future studies aimed at improving our understanding of hCMV-related hearing loss.

## Supporting information

S1 Fig Schematic illustration of the CMV glycoproteins and their interaction with host cell receptors. Created in BioRender. Grijpink, L. (2025) https://BioRender.com/c72j815
(JPEG)

S2 Fig Dot plot displaying standardized gene expression of host receptors and known marker genes in cochlear and vestibular cell types in human fetal inner ear tissue (FW7.5 and FW9.2). (TIF)

S3 Fig PDGFRA (magenta) protein expression in human fetal inner ear tissue (FW12.6) in the scala media. Nuclei were stained with DAPI. The dashed line indicates the mesothelial and epithelial cell layers that constitute Reissner’s membrane. PDGFRA^ +^ cells are present in the mesothelial layer of Reissner’s membrane and in the mesenchyme. Scale bar =  50 μm
(TIF)

S4 Fig PDGFRA (magenta) antibody compared to isotype control in the ampulla (FW8.6). Nuclei were stained with DAPI. (A) Immunofluorescent staining using a PDGFRA (magenta) antibody in the ampulla showing signal in the mesenchymal cells as well as the sensory domain. (B) Immunofluorescent staining using an isotype control showing signal in the sensory domain only.
(TIF)

S5 Fig NRP2 (cyan) protein expression in human fetal inner ear tissue (FW14.2) in the scala media. CD68 (yellow) and AIF1 (magenta) are markers for macrophages and show overlap with host receptor NRP2. Nuclei were stained with DAPI. SD =  sensory domain, HC =  hair cell, LDF =  lateral duct floor. (A) Mesenchyme lining the lateral duct floor containing two CD68^ + ^AIF1^ + ^NRP2^ +^ cells (arrowheads). (A”’) shows the merged images. (B) Cluster of CD68^ + ^AIF1^ + ^NRP2^ +^ on Reissner’s membrane (arrowhead) and a single cell in the mesenchyme (arrow). (B”’) shows the merged images. Scale bar =  50 μm.
(TIF)

S6 Fig NRP2 (cyan) protein expression in human fetal inner ear tissue (FW11.5). PECAM1 (yellow) is a marker for endothelial cells and MLANA (magenta) for melanocytes. Nuclei were stained with DAPI. (A) Capillary, scale bar =  10 μm. (B) Stria vascularis, scale bar =  20 μm. Arrows indicate MLANA^+^NRP2^-^ cells and the arrow head indicates a MLANA^+^NRP2^ +^ cell.
(TIF)
