# Supplementary figures and images for: Cytomegalovirus host receptor expression in the human fetal inner ear
Source: PLoS One. 2025 Mar 31;20(3):e0320605. doi: 10.1371/journal.pone.0320605 (PMC11957294; doi:10.1371/journal.pone.0320605)

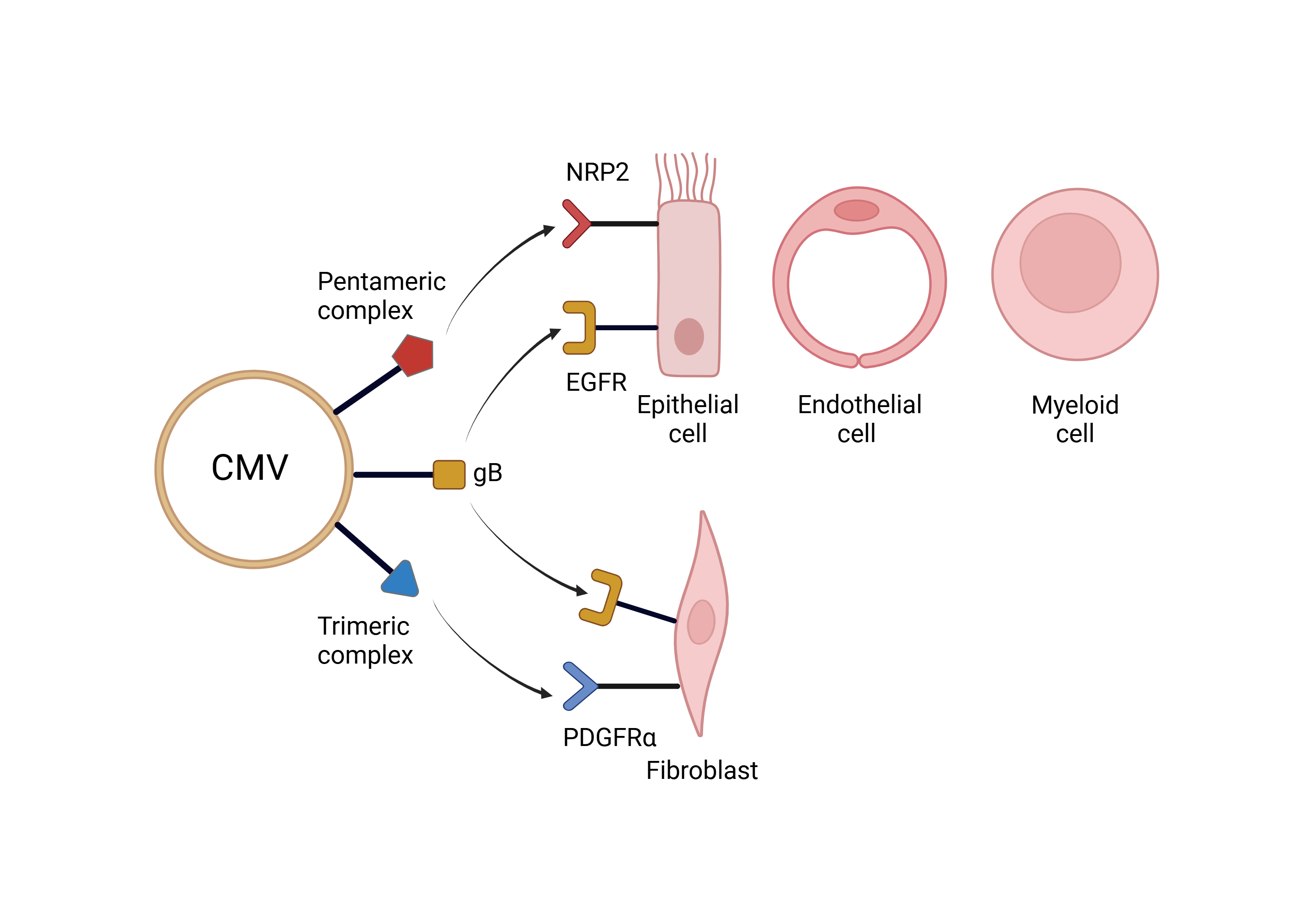

Supplement: S1 Fig — Created in BioRender. Grijpink, L. (2025) https://BioRender.com/c72j815 (JPEG) [file pone.0320605.s001.jpeg]

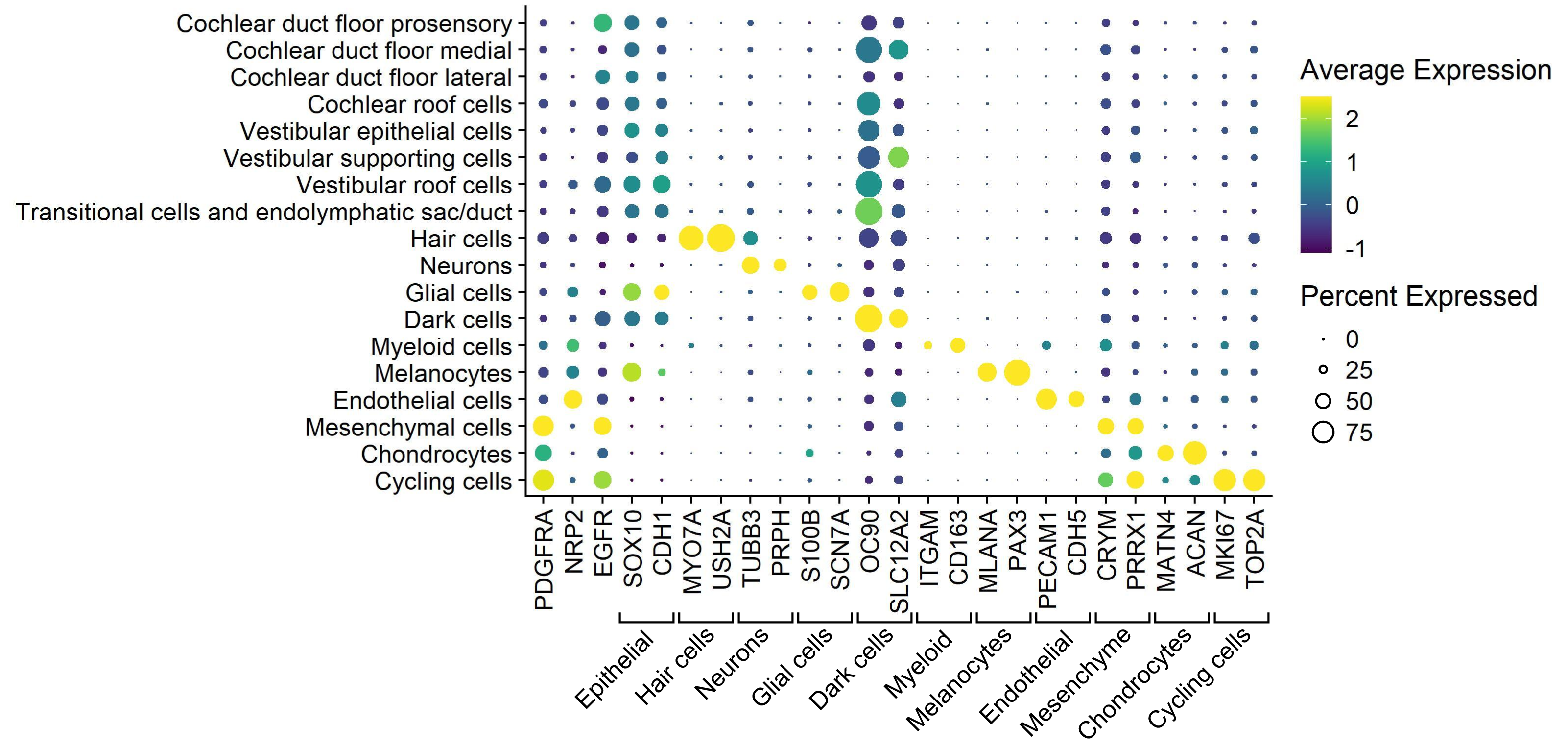

Supplement: S2 Fig — (TIF) [file pone.0320605.s002.tif]

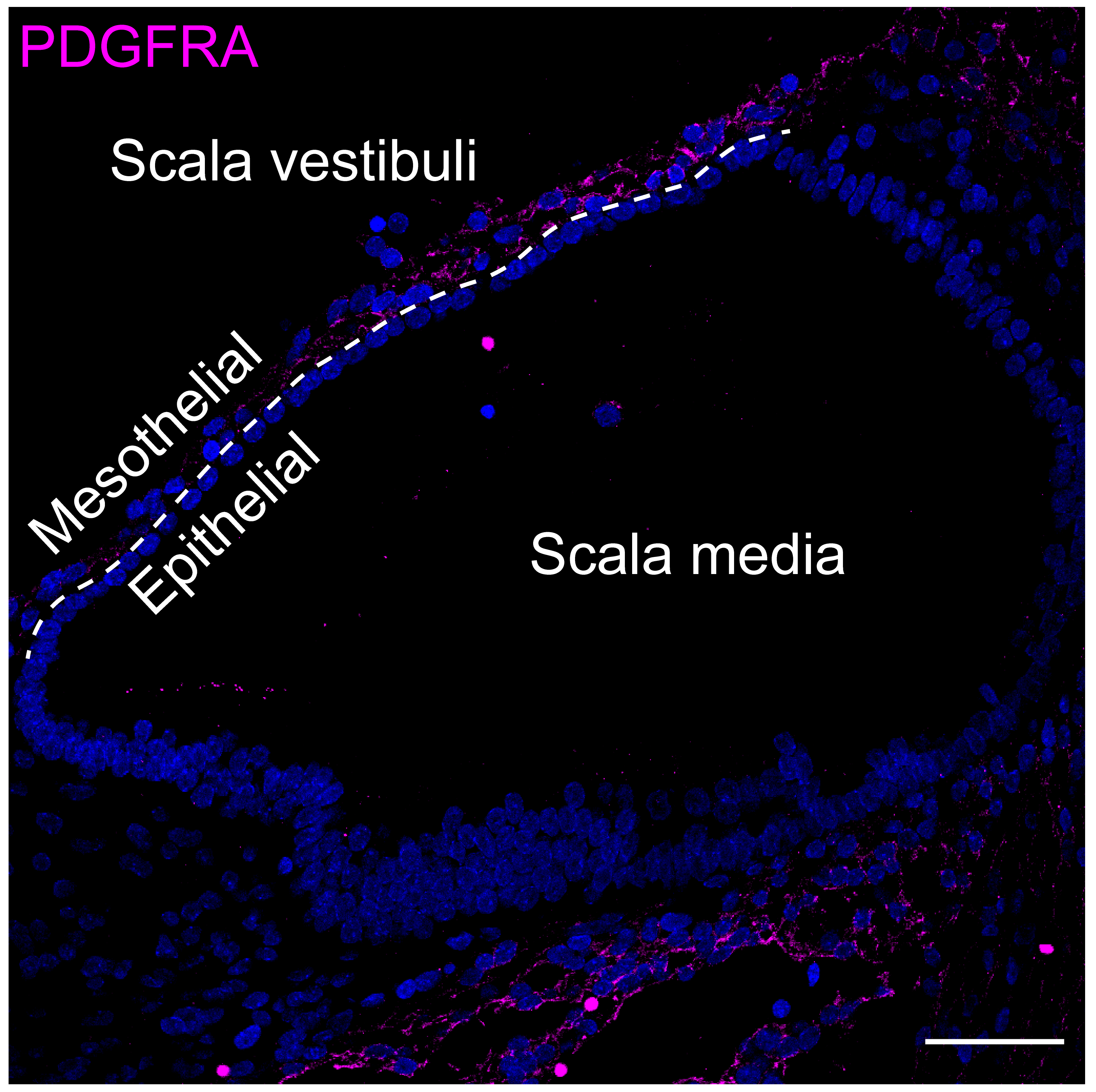

Supplement: S3 Fig — Nuclei were stained with DAPI. The dashed line indicates the mesothelial and epithelial cell layers that constitute Reissner’s membrane. PDGFRA + cells are present in the mesothelial layer of Reissner’s membrane and in the mesenchyme. Scale bar = 50 μm (TIF) [file pone.0320605.s003.tif]

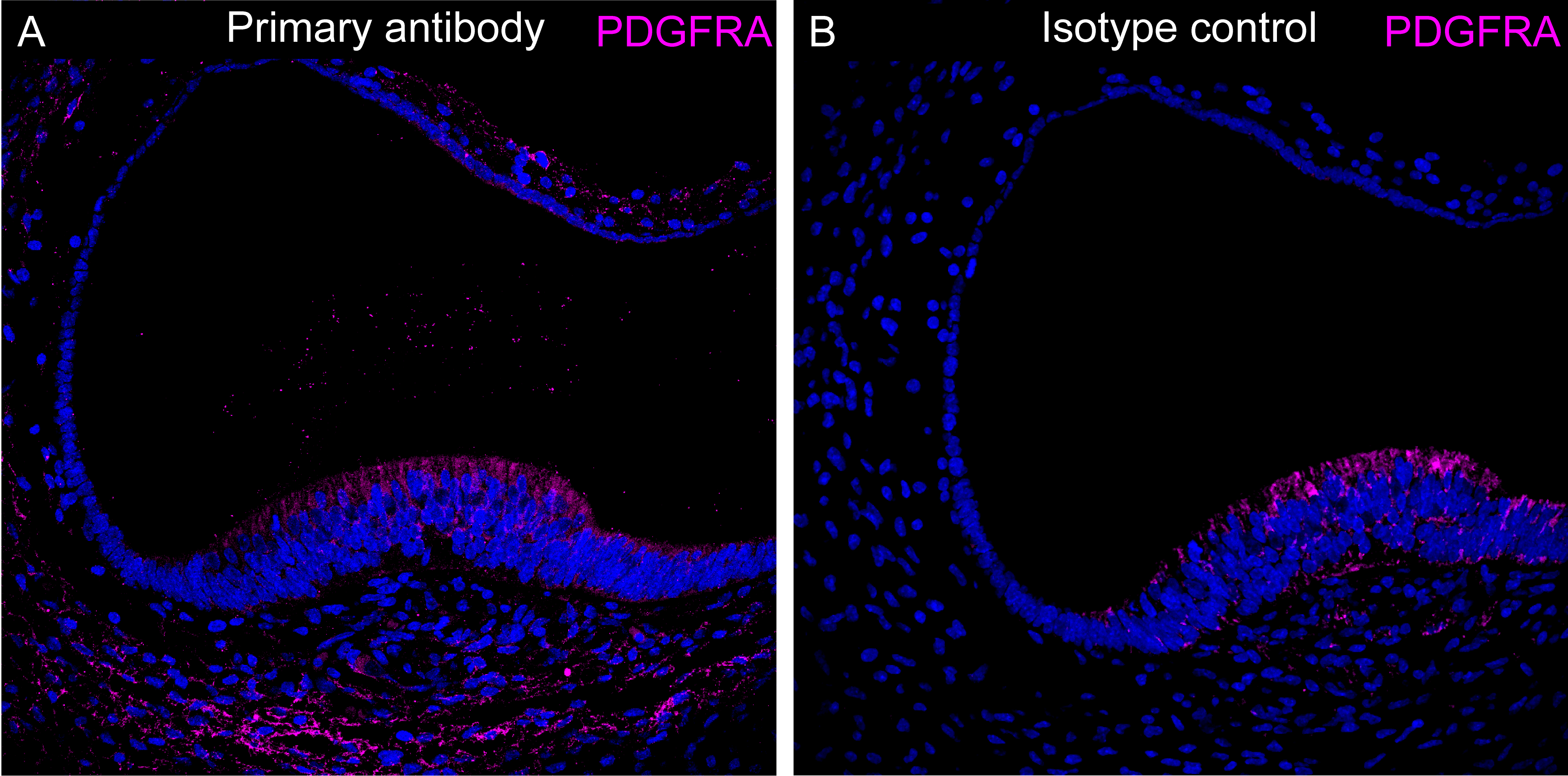

Supplement: S4 Fig — Nuclei were stained with DAPI. (A) Immunofluorescent staining using a PDGFRA (magenta) antibody in the ampulla showing signal in the mesenchymal cells as well as the sensory domain. (B) Immunofluorescent staining using an isotype control showing signal in the sensory domain only. (TIF) [file pone.0320605.s004.tif]

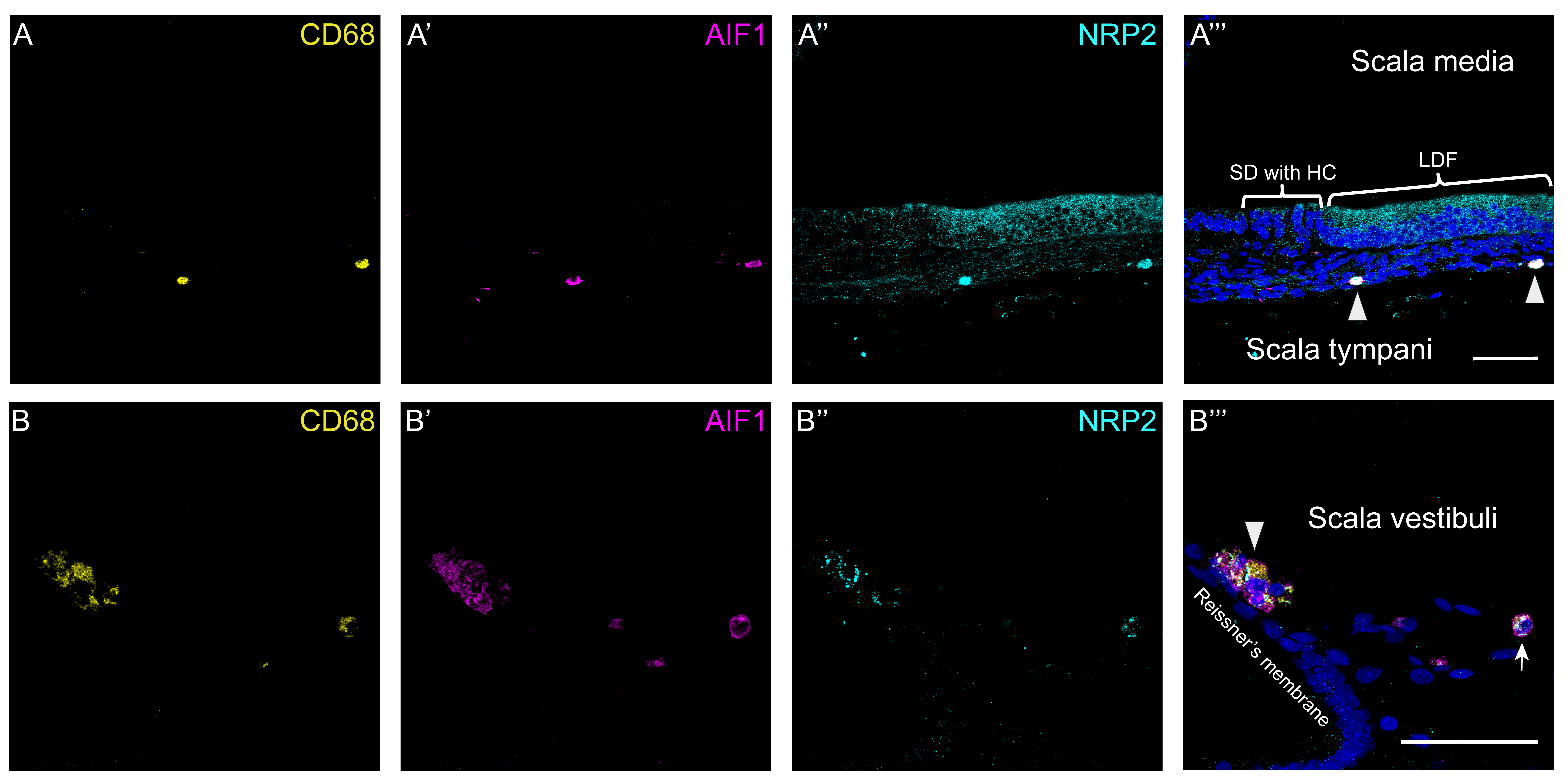

Supplement: S5 Fig — CD68 (yellow) and AIF1 (magenta) are markers for macrophages and show overlap with host receptor NRP2. Nuclei were stained with DAPI. SD = sensory domain, HC = hair cell, LDF = lateral duct floor. (A) Mesenchyme lining the lateral duct floor containing two CD68 + AIF1 + NRP2 + cells (arrowheads). (A”’) shows the merged images. (B) Cluster of CD68 + AIF1 + NRP2 + on Reissner’s membrane (arrowhead) and a single cell in the mesenchyme (arrow). (B”’) shows the merged images. Scale bar = 50 μm. (TIF) [file pone.0320605.s005.tif]

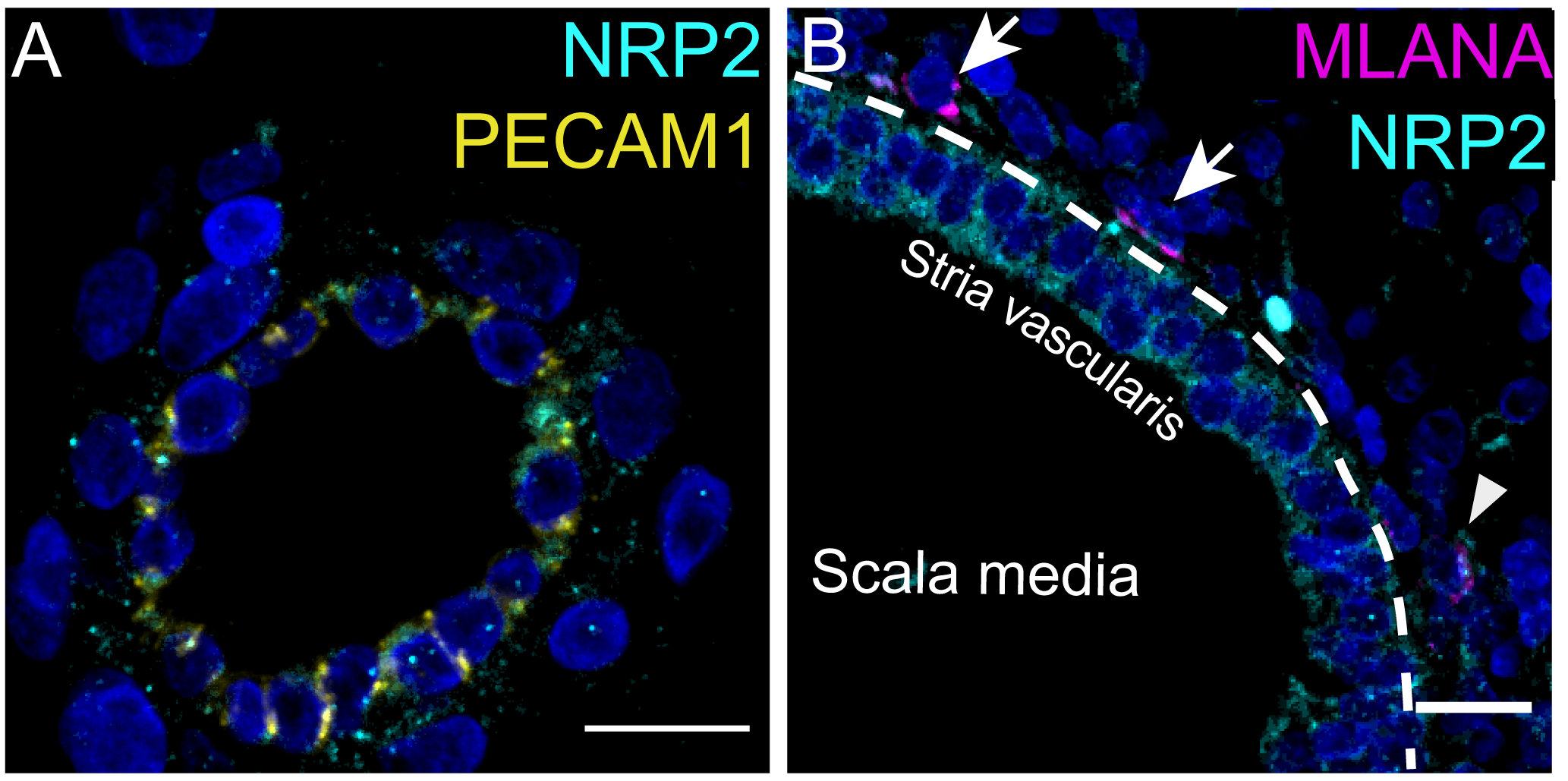

Supplement: S6 Fig — PECAM1 (yellow) is a marker for endothelial cells and MLANA (magenta) for melanocytes. Nuclei were stained with DAPI. (A) Capillary, scale bar = 10 μm. (B) Stria vascularis, scale bar = 20 μm. Arrows indicate MLANA+NRP2- cells and the arrow head indicates a MLANA+NRP2 + cell. (TIF) [file pone.0320605.s006.tif]
